# Highlights from Heart Rhythm 2018: Innovative Techniques

**DOI:** 10.19102/icrm.2018.090905

**Published:** 2018-09-15

**Authors:** Alan Sugrue, Samuel J. Asirvatham

**Affiliations:** ^1^Department of Cardiovascular Diseases, Division of Heart Rhythm Services, Mayo Clinic, Rochester MN, USA; ^2^Department of Pediatric and Adolescent Medicine, Division of Pediatric Cardiology, Mayo Clinic, Rochester MN, USA

**Keywords:** Artificial intelligence, atrial fibrillation, electroporation

In the following article, we share our insights from the exceedingly successful 2018 Heart Rhythm Society (HRS) meeting. As always, there is a tremendous amount of innovation at these meetings that touches upon many areas of electrophysiology. Although there are many outstanding presentations and abstracts, we have selected the following research abstracts from across a range of different presentation formats, as we feel that these highlight key innovation takeaways from the HRS conference.

## Beyond radiofrequency: the continued search for the ideal energy for ablation

The evolution and transformation of cardiac ablation has tracked a remarkable course over the last 50 years. In particular, the methods for cardiac ablation have tremendously advanced from the initial surgical approaches, which focused on ligation of the atrioventricular node,^1^ to the first use of direct current from a transvenous approach^2^ and the subsequent emergence and dominance of radiofrequency (RF) ablation. More recently, a desire has arisen to explore other potential ablation energy modalities such as microwave, laser, electric fields, and ultrasound. Today, RF is the most commonly used ablation energy, and although it is generally efficacious in destroying arrhythmogenic tissue, there is growing recognition of the shortcomings and complications of RF energy application, largely resulting from thermal heat generation. Particular concerns include collateral tissue damage (eg, to the phrenic nerve^3^ and esophagus^4,5^); coagulation formation; and silent cerebral infarcts/lesions and the long-term consequences of these.^6^ As such, alternative energy sources for ablation using microwave, laser, electric fields, and ultrasound have been studied with varying degrees of success. This year at HRS, we saw some noteworthy papers on different ablation energies as well as the first in-human data on the use of pulsed electric fields to ablate cardiac tissue.

### The first in-human application of pulsed electric fields

The first in-human data regarding the application of pulsed electric fields as a cardiac ablation modality was presented at HRS this year. The term “pulsed electric field” (also known as electroporation) refers to an ablation modality that employs high voltages, usually from direct current, that are delivered between two or more electrodes to create an electric field. Exposure of tissue to pulsed electric fields induces cell membrane changes, which result in the formation of pores. This formation of pores can be reversible or irreversible. The application of irreversible electroporation (IRE) as an ablation modality has been well studied in solid tumors and is an established United States Food and Drug Administration-approved treatment.^7–10^ It represents an alluring method for cardiac ablation, as it is considered nonthermal and can be tissue-specific and performed quickly. Although there has been significant work completed in animal models^11–18^ showing reasonable efficacy and safety with IRE ablation, this is the first in-human data.

In this late-breaking clinical trial by Reddy et al.,^19,20^ the investigators report the first acute clinical experience of atrial fibrillation ablation with pulsed electric fields in 22 patients using both a catheter-based approach (15 patients) and an epicardial approach (seven patients) during cardiac surgery. In those patients who were treated with the catheter-based approach, the total procedure time ranged from 52 minutes to 84 minutes, with pulsed electric field energy delivery time being less than 60 seconds per patient. The pulmonary veins (PVs) were isolated successfully in all patients. Meanwhile, in those who were treated with the surgical epicardial approach, pulsed electric field ablation was successful in six out of the seven patients, with the catheter time for epicardial ablation ranging from 30 minutes to 75 minutes. From a safety perspective, there were no related procedural complications or adverse events reported.

These critical human safety and acute efficacy data are very encouraging with respect to the ability of electric fields to be used as an ablation modality. Further research with chronic long-term follow-up and ultimately randomization against RF will be essential, but, overall, these findings indicate significant promise for the future of IRE.

### Epicardial microwave ablation

Microwave energy, as an alternative ablation modality, has also attracted much interest and could potentially overcome some of the limitations of RF. However, its use is still mainly undergoing clinical investigation. Microwave is an attractive modality, as, since thermal lesion development results from radiant heating, there is no requirement for catheter contact and the effect is not limited by electrode size. Furthermore, microwave radiation is not absorbed by the blood and has the benefit of limited heating of the endocardial surface, thereby restricting coagulum formation.^21^ Data at this year’s HRS conference show a potential use of microwave ablation to form deep lesions through epicardial fat without causing acute injury to nearby coronary vessels. Epicardial ablation is a critical approach in the treatment of arrhythmogenic substrate in specific cardiac pathologies. However, safety concerns—in particular, damage to the coronary vessels—represent a real issue, with angiography performed before and after ablation in high-risk areas with the overall goal of delivering ablation lesions at least 5 mm from a vessel. A method to perform epicardial ablation without concern for coronary artery vessel damage would be a well-received tool for the electrophysiologist.

In the presentation by Thomas et al.,^22^ the researchers highlighted a novel 9-French irrigated microwave ablation catheter that could potentially circumvent the issues with RF application in the epicardial space. In their study in sheep, the microwave catheter was positioned over the left ventricular summit, anterior interventricular groove, and left posterolateral ventricle close to the coronary vessels. Ablations of 90 watts (W) to 100 W at 20 mL/min for four minutes were delivered. There was no coronary vessel damage detected, and effective lesions were created. Although further studies, particularly in humans, will be required, this presentation highlights an innovative application beyond RF with the potential to enable the avoidance and mitigation of coronary vessel damage, particularly in the epicardial space.

### Laser

Laser ablation is another alternative ablation modality that produces lesions by inducing a vibrational excited state in molecules to create tissue heating and lesion formation. It can be delivered in either a continuous or pulse mode. To date, two generation of catheters have been produced, as the first-generation design had some significant limitations regarding its compliance and issues with thrombus formation due to direct laser absorption by the blood.^23^ The second-generation design has a bloodless interface, which transfers the laser energy through a compliant balloon. Generally, initial results have shown good short- and mid-term clinical outcomes in accordance with the results from RF ablation.

At HRS this year, we also saw the presentation of the five-year outcomes data with ablation using a visually guided laser balloon ablation (VGLB) procedure.^24^ In this study by Reissmann et al.,^24^ in 90 patients evaluated over a median follow-up of 36 months following a single VGLB procedure, the investigators reported that the five-year freedom from arrhythmia recurrence was 51%. Thirty-three patients underwent repeat RF ablation procedures, with major periprocedural complications occurring in four of them (4%).

Although from a small study cohort, these long-term data seem to suggest a high recurrence rate, particularly when compared to RF and cryotherapy at five years of follow-up. Nevertheless, there were also interesting data examining and comparing acute reconnection after PV isolation with laser-balloon versus RF ablation. Considering that the majority of AF reoccurrence is driven by PV reconnection and that the five-year arrhythmic-free survival was low, more PV reconnections possibly occur when using a laser. However, a careful study also presented at this year’s HRS conference by Uecer et al.^25^ noted that PVs isolated with VGLB were significantly less prone to reconnection as compared with those PVs isolated with RF energy [10 PVs isolated with VGLB (10.8%) versus 29 PVs isolated with RF energy (30.9%); p = 0.001]. Therefore, whether there is anything long-term that relates explicitly to laser ablation, particularly lesion formation, is unclear. Future publications and studies involving larger patient cohorts may help to provide further clarity.

## Ablation biophysics: high (or very-high)-power, short-duration ablation—a changing paradigm?

RF ablation historically has been delivered at a power level between 20 W and 40 W for a duration of 20 seconds to 40 seconds. As we discussed, there are many limitations with the use of RF energy, and challenges remain in the delivery of durable lesions. The incidence of PV reconnection also remains significant, and, although the cause of PV reconnection is multifactorial, some evidence points toward possible incomplete ablation lesion formation.^26^ Consequently, the concept of “high-power, short-duration (HP-SD)” ablation has been proposed as an alternative approach, with high power generally referring to a power of 50 W applied for five seconds to 10 seconds, or very high power (90 W) delivered for four seconds. The rationale for this HP-SD approach is the belief that this ablation strategy can create an immediate transmural injury, limiting the application time and risk of catheter motion. Further, the shorter duration limits passive heart conduction, which has the potential to avoid damage to collateral tissue. Ultimately, this approach increases resistance heating and reduces conductive heating. As we continue to improve, explore, and perfect ablation power strategies, it is important to note that there was a variety of well-presented abstracts at HRS this year on HP-SD ablation.

Regarding preclinical data with this approach, Nguyen et al.^27^ presented some interesting data from both ex vivo and in vivo models showing that HP-SD RF ablation had similar lesion volumes but less lesion depth in comparison with lower-power, longer-duration RF ablation. In addition, as compared with low-power, long-duration RF, tissue temperatures were similar, but overall temperature–time integrals were lower with the HP-SD approach. The investigators suggested that this approach might be useful in areas where shallow ablation may be required, such as the posterior atrium. In addition to this study, other data presented by Nakagawa et al.^28^ showed that the application of 90 W of power for four seconds and 50 W of power for 10 seconds during ablation produced similar maximum lesion diameters and surface diameters without increasing the risk of steam pop or thrombus. Related data were also presented by Ikeda et al.^29^

There were additionally two presentations on human data, with the first by Winkle et al.^30^ examining the complication rates from four experienced centers performing RF ablations for AF at powers from 45 W to 50 W for 10 seconds to 15 seconds. In 11,436 ablations using 45 W to 50 W of power on the posterior wall of the left atrium, death was recorded in two (0.014%) patients (one due to stroke and one due to ventricular tachycardia), pericardial tamponade in 33 (0.24%; 26 tapped and seven surgical), stroke occurring at < 48 hours postprocedure in six (0.043%), strokes occurring between 48 hours and 30 days postprocedure in six (0.043%), PV stenosis requiring intervention in two (0.014%), phrenic nerve paralysis occurring in two (0.014%; both resolved), steam pops presenting in two (0.014%), and atrioesophageal fistula occurring in one. The second notable paper, by Liu et al.,^31^ reported the achievement of safety and one-year efficacy at 50 W for five seconds to 10 seconds. They showed a low complication rate, and freedom from AF was comparable to ablation with standard power (20–40 W, 20–30 seconds) at one year.

Overall, these data are intriguing. As catheter technology continues to develop to deliver this energy optimally and we see more trials on this approach, we may soon view HP-SD ablation as the optimal setting to perform ablation, particularly in discrete areas of the heart such as the posterior wall.

## Artifical intelligence: what does the future hold?

Artificial intelligence (AI) is already deeply integrated into our lives and has shown tremendous promise in medicine. This year alone, the US Food and Drug Administration has approved three devices that use AI to help patients. The ability of AI to detect and recognize patterns that elude humans provides unique opportunities to further our understanding of disease pathophysiology, diagnosis, and treatment. Regarding cardiology, there is tremendous potential for AI, and we saw glimpses of it this year at HRS with the presentation of two notable papers in this area.

The first, by Bos et al.,^32^ applied AI to determine if it was better than the corrected QT interval alone at distinguishing patients with concealed long QT syndrome from healthy patients. This is an important question and one that we have tried to help address through the application of architectural T-wave analysis.^33^ In this presentation, however, Bos et al.^32^ took this analysis one step further and, with the application of deep neural networks in the analysis of surface ECGs, highlighted that they were able to successfully distinguish patients with electrocardiographically concealed long-QT syndrome from normal patients and provide a nearly 80% accurate pregenetic test anticipation of long-QT syndrome genotype status.

The second presentation, by Boleyn et al.,^34^ examined and reported on the development of an automatic screening technology to detect AF. Remarkably, the AI program was able to differentiate AF not only from normal sinus rhythm but also from other conditions that cause beat-to-beat variation such as dense ectopy, ectopic atrial rhythm, and atrial flutter with variable conduction conditions, which are commonly mistaken for AF using R–R variability algorithms. Further development of this software and evaluation of its benefit as a screening mechanism could potentially have a considerable impact upon the general population.

As showcased, the potential for AI is enormous. However, we need to be cautious with our application of AI. Unfortunately, AI is a “black box,” in that we do not know exactly how it reaches its decisions, and it subsequently can become biased if inadequate care is paid to the information it is learning from.

## Novel software and devices: pushing the limits of our technology

At HRS ever year, there is a vast array of innovative software or device technologies, some of which we have mentioned above and others we have not yet discussed. This year was no exception, and there were several abstracts that we felt were standouts in this area.

### Irrigated intramural needle

In the presentation by Stephenson et al.,^35^ the investigators discussed the safety and outcomes of patients who received ablation with an irrigated intramural needle. This novel technology is an essential addition to the electrophysiologists’ armamentarium, particularly for ventricular arrhythmias, as the intramural myocardium can be a crucial etiological site. Further, intramural sources are often a reason for ablation failures; therefore, a tool to target intramural sources safely and efficaciously is essential. In this late-breaking trial, an irrigated needle ablation was performed in 31 patients at three centers. With a median of 15 RF needle applications per patient delivered to selected right ventricular/left ventricular septum and free wall sites, needle ablation resulted in a substantial improvement in 68% of patients at six months of follow-up. Six patients experienced a complication from this approach. Overall, these data are attractive and have the potential to be a handy tool for the future electrophysiologist.

### Percutaneous transverse sinus epicardial pacing and defibrillation device

A novel method of epicardial pacing and defibrillation using the transverse sinus as an anchor point was proposed by Vaidya et al.^36,37^ Transvenous implantation of pacing or defibrillation leads is the most typical technique of lead placement. However, there are multiple potential drawbacks to this approach. The transverse sinus offers a unique opportunity to cross the heart from left to right and can be employed to help with the pass-through of an ablation or mapping catheter. It is therefore feasible that a percutaneously placed epicardial device lead could utilize this space. With this in mind, the authors designed a novel epicardial pacing and defibrillation device. In a preclinical model, placement of the transverse lead was successful without any compilations or adverse events. Capture was feasible without extracardiac stimulation. Mean pacing thresholds were 0.77 mV ± 0.55 mV for the atrium and 1.4 mV ± 1.6 mV for the ventricle. Defibrillation was successfully delivered at seven of 10 sites (70%), with an average defibrillation threshold of 11 J. Although further chronic studies will be required, this highlights a novel unique approach.

### Novel three-dimensional technology for both fluoroscopy and visualization of cryoballoon catheters

As cardiology continues to develop and test more complex systems for mapping and ablation, the integration of these systems with current technology and each other will become critically important. In two abstracts by Bourier et al.^38,39^ presented at this year’s HRS conference, the authors gave us a glimpse of what this integration may look like. In the first presentation, they combined a three-dimensional (3D) mapping system with computed tomography images to enable the creation of 3D-augmented fluoroscopy. 3D-overlay contour offsets were 3.1 mm ± 2.8 mm and the average time needed for anatomy segmentation was 200 seconds ± 80 seconds (preprocedural), with the time required for overlay registration being 25 seconds ± 14 seconds (intraprocedural). In the second abstract, the first technology for 3D visualization of cryoballoon catheters was presented. Although the 3D technology is available for RF visualization at present, there is nothing on the market yet available for cryoballoon technology. Using custom software, the authors demonstrated that they could calculate a virtual 3D representation of the cryoballoon, enabling it to be visualized on fluoroscopy. The accuracy of reconstruction was 2.0 mm ± 0.4 mm (n = 100). These papers give us a glimpse of the future electrophysiology laboratory, where multiple advanced systems will be combined to provide the operator with both virtual and augmented reality.

In conclusion, the 2018 HRS meeting truly showcased some remarkably innovative techniques and advancements. We eagerly look forward to seeing what next year has to offer.
